# Novel Mastadenovirus Infection as Cause of Pneumonia in Imported Black-and-White Colobuses (*Colobus guereza*), Thailand

**DOI:** 10.3201/eid3012.241042

**Published:** 2024-12

**Authors:** Chutchai Piewbang, Sabrina Wahyu Wardhani, Panida Poonsin, Pattiya Lohavicharn, Ratchanon Tengtawon, Thanakrit Charoenrat, Sitthichok Lacharoje, Sawang Kesdangsakonwut, Tanit Kasantikul, Nathamon Kosoltanapiwat, Somporn Techangamsuwan

**Affiliations:** Chulalongkorn University, Bangkok, Thailand (C. Piewbang, S.W. Wardhani, P. Poonsin, P. Lohavicharn, S. Lacharoje, S. Kesdangsakonwut, S. Techangamsuwan); Goodwill Animal Hospital, Nonthaburi, Thailand (R. Tengtawon, T. Charoenrat,); Michigan State University, East Lansing, Michigan, USA. (T. Kasantikul); Mahidol University, Bangkok (N. Kosoltanapiwat)

**Keywords:** Adenovirus, viruses, respiratory infections, zoonoses, colobus, mastadenovirus, old-world monkey, nonhuman primates, pneumonia, Thailand

## Abstract

We identified a novel mastadenovirus, herein referred to as colobus adenovirus (CoAdV), as the likely cause of fatal respiratory and enteric diseases in multiple black-and-white colobuses (*Colobus guereza*) imported into Thailand in 2022. Among 9 affected colobuses, 4 died. Viral antigen was abundant in respiratory and enteric tissues, where prominent lesions and clinical signs were observed. We successfully cultivated CoAdV in Vero cells and characterized the complete viral genome, which indicated the virus is genetically distinct from other simian adenoviruses. We also conducted a retrospective study of archival samples from 7 other unrelated colobuses that had respiratory distress or diarrhea and found similar viral strains in 4 of those colobuses. Although we could not determine the potential harm to humans or other nonhuman primates from current information, the zoonotic and spillover potential of this virus to other related hosts should not be neglected. Veterinarians should consider CoAdV when pneumonia is diagnosed in colobuses.

Most adenoviruses that infect mammals belong to the genus *Mastadenovirus*, which is part of the family Adenoviridae and includes a variety of viruses found in humans, nonhuman primates (NHPs), and other mammals (*1*,*2*). Although many cases of human adenovirus infection are predominantly asymptomatic and infection can be self-limited in immunocompetent hosts, infections in immunosuppressed patients can lead to greater severity and even death (*3*–*6*).

Of the >50 adenovirus species in the genus *Mastadenovirus*, 9 that infect NHPs, known as simian adenoviruses (SAdVs; SAdV-A–I), and 7 that infect humans, known as human adenoviruses (HAdVs; HAdV-A–G), have been documented (*1*). Although species domination is considered on the basis of infected hosts, many adenoviruses found in NHPs have been identified as HAdVs (*7*), and SAdV infections have also been reported in humans who have contact with NHPs (*6*). Because of the phylogenetic proximity between NHPs and humans, interspecies transmission is highly possible (*8*,*9*). Although adenoviruses have been considered host-specific and restricted to >1 related species (*4*), phylogenetic and evolutionary analyses reveal instances of interspecies transmission that have led to new host-virus adaptations. For example, HAdV-E is believed to have originated from chimpanzee adenoviruses and HAdV-B from gorilla adenoviruses (*10*). In addition, serologic evidence indicates that adenoviruses derived from monkeys can infect humans who have close contact with NHPs (*5*,*6*). Those findings raise concerns about interspecies transmission and zoonotic potential. Notable examples include canine adenovirus 1, which infects multiple carnivore species, and skunk adenovirus 1, found in marmosets (*Callithrix* spp.), skunks (*Mephitis* spp.), pygmy hedgehogs (*Atelerix albiventris*), and other species, illustrating adenoviruses’ ability to cross species barriers (*11*,*12*). Such instances underscore the potential zoonotic threats posed by mastadenoviruses because of their genetic diversity and adaptability (*13*).

Southeast Asia and China are main hubs of the primate trade, both as importers and exporters, for various purposes (*14*,*15*). Importation of apparently healthy NHPs without specific surveillance for NHP-related adenoviruses may result in the introduction of novel adenoviruses to naive environments or vice versa. We identified a novel adenovirus in colobuses with fatal respiratory distress that were imported into Thailand in 2022. 

## Materials and Methods

During August–September 2022, nine wild-caught adult black-and-white colobuses (*Colobus guereza*) of unknown specific ages were imported from South Africa and kept as pets in individual stainless-steel cages in Bangkok, Thailand. All animals were shipped together in a single lot and underwent quarantine at customs for several months before all 9 went to a single pet owner. Initially, clinical signs and symptoms began with depression, reduced appetite, and fever, after which coughing, pneumonia, and subsequent respiratory distress developed. Those symptoms were observed shortly (within a few weeks) after the animals arrived at the final destinations with the pet owner. Four of the colobuses (nos. 1–4) succumbed to their illnesses 5–10 days after the onset of clinical symptoms; the other 5 (nos. 5–9) survived after receiving intensive supportive care.

Clinical samples, including oral and rectal swab samples, were collected from all 9 diseased colobuses. Among the 9 colobuses, the 4 that subsequently died (nos. 1­–4) were submitted for necropsy at the Department of Pathology, Faculty of Veterinary Science, Chulalongkorn University, Bangkok. We collected vital samples, including brain, trachea, lung, heart, intestine, liver, kidneys, and lymph nodes, and divided samples into 2 cohorts: fresh-frozen tissues and formalin-fixed paraffin-embedded (FFPE) tissues fixed in 10% buffered formalin. This study was approved by Chulalongkorn University Animal Care and Use Committee (approval no. 2431048).

### Nucleic Acid Extraction and Routine Diagnostic Panels

We processed collected samples for viral nucleic acid extraction by using QIAamp Viral RNA Mini Kit (QIAGEN, https://www.qiagen.com) according to the manufacturer’s guidelines. To elucidate the viral infections in these cases, we subjected the extracted nucleic acid samples to routine virologic testing panels by using pan-family virologic PCRs targeting Herpesviridae (*16*), Paramyxoviridae (*17*), Pneumoviridae (*17*), Parvoviridae (*18*), Orthomyxoviridae (*19*), Retroviridae (*20*), and Adenoviridae (*21*) families. For PCR-positive samples, we sent target amplicons to Celemics, Inc. (https://www.celemics.com) for next-generation sequencing (NGS)–based methods. Furthermore, we submitted fresh lung samples from all investigated colobuses for aerobic bacterial culture.

### Detection of Adenovirus DNA and Complete Genome Characterization

For samples that initially tested positive for adenoviruses by pan-Adenoviridae PCR targeting the DNA polymerase (*DNApol*) gene, we reconfirmed results by using a second PCR that targeted the *hexon* gene with established pan-*Mastadenovirus* primers (*22*). We then sequenced the positive amplicons following the protocol described previously. We used nucleic acid extracts obtained from fresh lung tissue of colobus no. 1 for complete adenoviral genome characterization through a de novo*–*based NGS method (Vishuo Biomedical, https://www.vishuo.com) (Appendix). Subsequently, we aligned the identified sequence with previously described adenovirus sequences found in NHPs, including SAdV-A–I and unclassified SAdVs. We subjected that alignment to maximum-likelihood phylogenetic analysis (Appendix). We also performed recombination analysis by using a recombination detection program (*23*) (Appendix). We refer to this novel virus as colobus adenovirus (CoAdV).

### Viral Load Quantification and Localization

To confirm the results from NGS analysis and investigate the genomic distribution of CoAdV in various organs and in other colobuses, we conducted conventional PCR using newly designed primers targeting the *DNApol* gene of CoAdV. In addition, to quantify viral loads in different organs, we performed a SYBR green–based quantitative PCR (qPCR) by using KAPA SYBR Fast qPCR Master Mix (2X) Universal Kit (Sigma-Aldrich, https://www.sigmaaldrich.com) with specific primers designed to target the *DNApol* gene of CoAdV (CoAdV-DpolF-6348, 5′-CAG CTG GTC CTC GTC C-3′; CoAdV-DpolR-6443, 5′-TTG CAG GAC CCC CTG AAG AC-3′). We estimated relative viral loads in each organ on the basis of cycle threshold (Ct) values.

### Detection of CoAdV Antigen in FFPE Tissues

To determine the tissue tropism of CoAdV, we subjected FFPE tissues from the 4 necropsied colobuses that tested positive in both conventional and qPCR to immunohistochemical (IHC) analysis. The examined tissues were brain, lung, liver, lymph node, spleen, heart, kidneys, and intestine. We assessed localization of CoAdV in those tissues by using a horseradish peroxidase polymer-conjugated method with primary antibody against adenovirus clones 2/6 and 20/11 (Chemi-Con, https://chemi-con.com) (Appendix).

### Viral Isolation

We conducted virus isolation on Vero cells (ATCC accession no. CCL-81) in 12-well tissue culture plates. We subjected samples to viral isolation, including oral and rectal swab samples from diseased colobus nos. 4–6, 7, and 9 that had been collected in viral transport medium, and supernatants from lung and trachea homogenized in PBS from colobus no. 1 that died (Appendix). We monitored cultures for cytopathic effects (CPE) daily for 5 days (*2*). We collected supernatants from wells showing CPE and stored at –80°C for subsequent adenovirus-specific PCR confirmation. In addition, we harvested cell pellets and prepared for transmission electron microscopy analysis.

### Retrospective Study of CoAdV in Other NHPs

We also used the qPCR described to conduct CoAdV detection on a small sample cohort that underwent postmortem examination at Department of Pathology, Faculty of Veterinary Science, Chulalongkorn University, during 2020–2022. The cohort comprised archival respiratory or rectal swab samples and fresh-frozen lung tissues obtained from 7 other colobuses that died from various causes, plus the fresh-frozen lung or respiratory swab samples from other primates, including 2 Douc monkeys (*Pygathrix* spp.), 1 De Brazza’s monkey (*Cercopithecus neglectus*), 6 marmosets, 4 Japanese macaques (*Macaca fuscata*), 2 cotton-top tamarins (*Saguinus oedipus*), and 11 long-tailed macaques (*Macaca fascicularis*).

## Results

### CoAdV Detection, Genome Characterization, and Phylogenetic Analysis 

Apart from negative results of ancillary testing using pan-family virologic PCR panels, the oral swab samples from all 9 colobuses and 5 fecal swab samples obtained from colobuses nos. 1–4 and 7 tested positive by pan-adenovirus PCR. The colobuses that subsequently died (nos. 1–4) predominantly exhibited lower Ct values (Table). Analysis of tissue samples showed that the lung, trachea, and intestines exhibited positive amplification signals for the pan-adenovirus PCR, whereas the other collected tissues had negative results. *Klebsiella* spp. was cultivated from the lung tissue of colobus nos. 1 and 4. We sequenced the positive adenovirus amplicons (460 bp) from all samples, and initial BLASTn (https://blast.ncbi.nlm.nih.gov) results showed 99.9% nucleotide similarities among adenovirus from colobuses and most were closely related to another SAdV (GenBank accession no. JN808448).

**Table:** Immunohistochemistry and quantitative PCR findings in an investigation of novel mastadenovirus infection causing pneumonia in imported black-and-white colobuses (*Colobus guereza*), Thailand*

Colobus no.	Sample types tested, Ct value/immunohistochemistry findings										
	Oral swab	Fecal swab	Lung	Trachea	TLN	Intestine	MLN	Liver	Kidney	Heart	Brain
1†	22.5/ND	30.2/ND	18.2/++	20.2/++	30.6/+	29.0/++	−/−	−/−	−/−	−/−	−/−
2†	23.4/ND	32.5)/ND	20.2/++	24.2/+	32.7/–	30.6/+	−/−	−/−	−/−	−/−	−/−
3†	26.9/ND	32.8/ND	22.4/++	23.8/++	30.2/+	30.2/+	−/−	−/−	−/−	−/−	−/−
4†	22.8/ND	29.9)/ND	17.5/++	24.9/++	33.8/–	25.8/+	−/−	−/−	−/−	−/−	−/−
5	29.0/ND	­–/ND	NSA	NSA	NSA	NSA	NSA	NSA	NSA	NSA	NSA
6	29.8/ND	–/ND	NSA	NSA	NSA	NSA	NSA	NSA	NSA	NSA	NSA
7	30.5/ND	34.5/ND	NSA	NSA	NSA	NSA	NSA	NSA	NSA	NSA	NSA
8	27.2/ND	–/ND	NSA	NSA	NSA	NSA	NSA	NSA	NSA	NSA	NSA
9	29.3/ND	–/ND	NSA	NSA	NSA	NSA	NSA	NSA	NSA	NSA	NSA

*Staining intensity of immunohistochemistry was semiquantitative. Ct, cycle threshold; MLN, mesenteric lymph node; ND, not determined; NSA, no sample available; TLN, tracheobronchial lymph node; +, positive intensity; ++, strong positive intensity; –, negative. †Colobus died.

**Figure 1:**
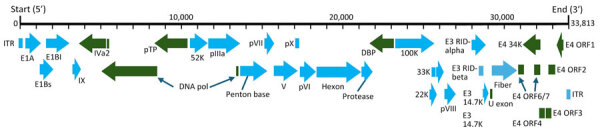
Genome organization of novel mastadenovirus infection causing pneumonia in imported black-and-white colobuses (*Colobus guereza*), Thailand. Thick black line indicates virus genome; gene length is also indicated. ITR sequences and putative viral genes are represented as rectangles or arrows, corresponding to the transcriptional direction. Within the rectangles or arrows, light blue color indicates 5′ to 3′ translational direction, and dark green indicates complementary translational direction. The virus, tentatively named colobus adenovirus, is available in GenBank (accession no. PP985428). DBP, DNA-binding protein; E, early region; ITR, inverted terminal repeat; ORF, open reading frame; RID, receptor internalization and degradation.

NGS results showed a total of 889 sequence reads that mapped to various simian mastadenovirus species. From those sequence reads, we generated a consensus sequence of 33,813 bp, tentatively named CoAdV strain CP001-TH/2023. To confirm the accuracy of the generated CoAdV sequence, we used primers for adenovirus screening as described and submitted the 460-bp PCR products for Sanger sequencing, which showed 100% similarity to the generated sequence. The CoAdV genome comprises 19.85% A, 18.34% T, 30.80% C, and 31.01% G. The CoAdV strain CP001-TH/2023 genome contained 145-bp ends of inverted terminal repeats with a conserved motif CATCATCCAAT, displaying typical mastadenovirus orthologs. The CoAdV CP001-TH/2023 genome possessed 32 predicted proteins and included genus-specific genes encoding proteins V and IX and the early (E) regions E1, E3, and E4. Only one *fiber* gene was observed within the CoAdV CP001-TH/2023 genome (Figure 1). We submitted the CoAdV sequence to GenBank (accession no. PP985428).

**Figure 2:**
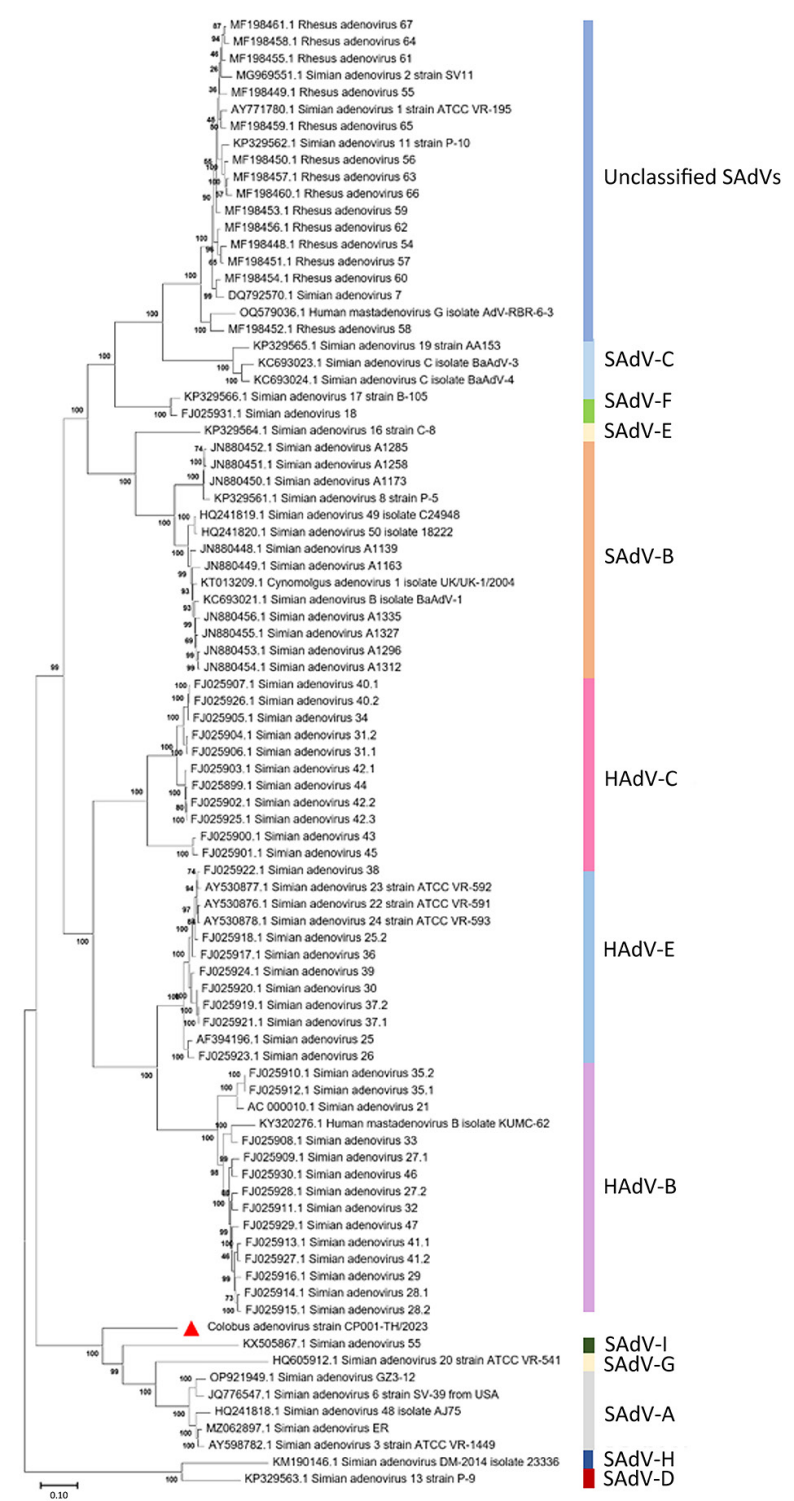
Phylogenetic analysis of complete genome sequence of novel mastadenovirus infection causing pneumonia in imported black-and-white colobuses (*Colobus guereza*), Thailand. Comparative analysis of the genome from this study, tentatively name colobus adenovirus (CoAdV), with various adenoviruses identified in nonhuman primates. Bootstrap values are shown at each node. Red triangle indicates the CoAdV CP001 TH/2023 identified in this study, and phylogenetic reveals this sequence groups with various SAdVs and HAdVs. GenBank accession numbers are provided. Scale bar indicates number of substitutions per site. HAdV, human adenovirus; SAdV, simian adenovirus.

**Figure 3:**
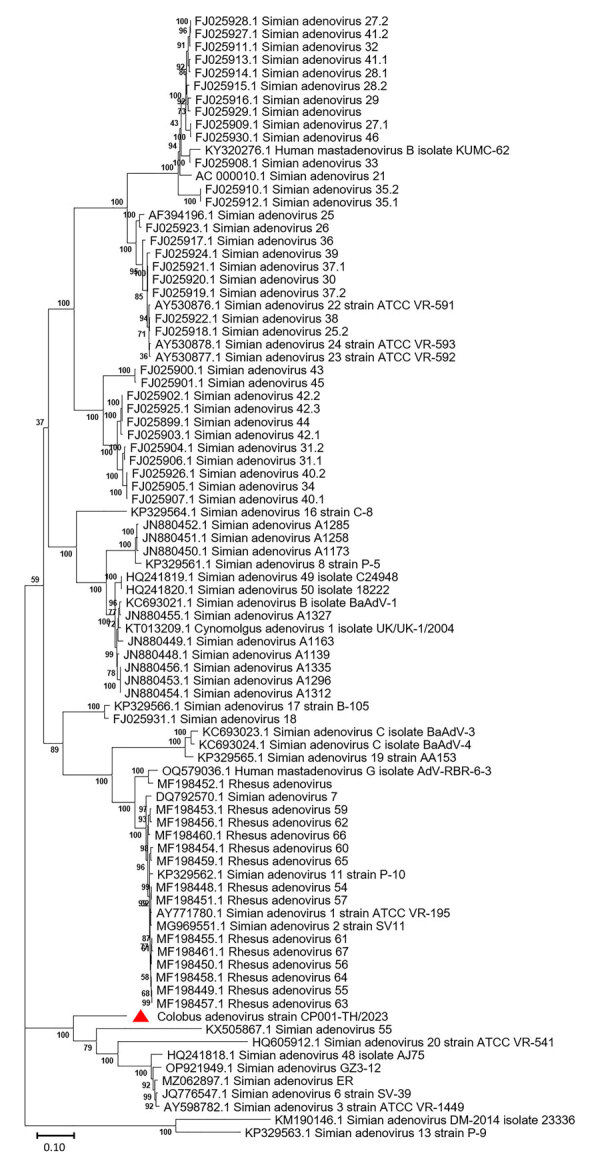
Phylogenetic analysis of amino acid sequences of *DNApol* gene of novel mastadenovirus infection causing pneumonia in imported black-and-white colobuses (*Colobus guereza*), Thailand. Comparative analysis of the genome from this study, tentatively name colobus adenovirus (CoAdV), with various adenoviruses identified in nonhuman primates. Bootstrap values are shown at each node. Red triangle indicates the CoAdV CP001 TH/2023 identified in this study. GenBank accession numbers are provided. Scale bar indicates the number of substitutions per site. AdV, adenovirus; BaAdV, baboon adenovirus.

**Figure 4:**
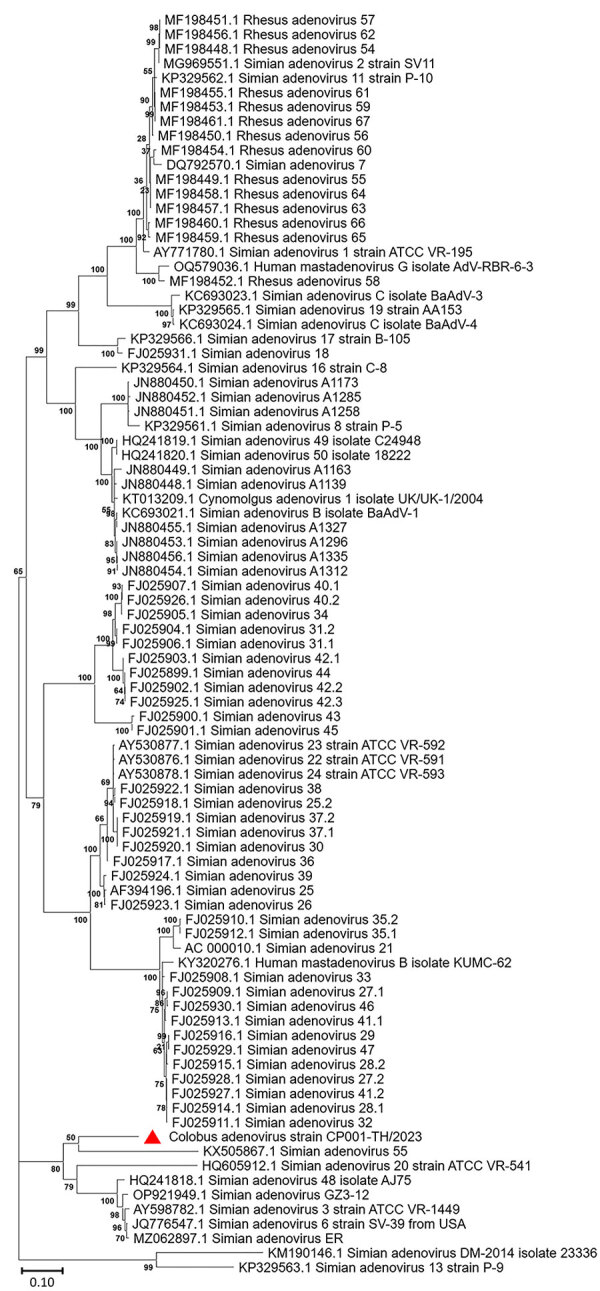
Phylogenetic analysis of amino acid sequences of *IVa2* gene of novel mastadenovirus infection causing pneumonia in imported black-and-white colobuses (*Colobus guereza*), Thailand. Comparative analysis of the genome from this study, tentatively name colobus adenovirus (CoAdV), with various adenoviruses identified in nonhuman primates. Bootstrap values are shown at each node. Red triangle indicates the CoAdV CP001 TH/2023 identified in this study. GenBank accession numbers are provided. Scale bar indicates the number of substitutions per site. AdV, adenovirus; BaAdV, baboon adenovirus.

The full-length CoAdV strain CP001-TH/2023 exhibited 67.10% nucleotide similarity to the SAdV-6 strain SV-39 (GenBank accession no. JQ776547) from Old World macaque monkeys in the United States. CoAdV CP001-TH/2023 was distinct from other SAdVs, and nucleotide similarities ranged from 66.50% to 50.02%. The human mastadenovirus B isolate KUMC-62 (GenBank accession no. KY320276) was the most distant strain, showing only 50.02% nucleotide similarity to the detected CoAdV. Analysis of *DNApol* gene revealed nucleotide similarities of 62.30%–77.90% and amino acid similarities of 25.40%–57.6% to previously described SAdVs. The SAdV strain ER (GenBank accession no. MZ062897) identified in China was the most genetically similar strain. Phylogenetic analysis of the full-length genome of SAdVs revealed that CoAdV CP001-TH/2023 formed a distinct phylogenetic cluster, and was most closely related to SAdV-A, SAdV-G, and SAdV-I isolated from Old World monkeys (Figure 2) and that SAdV-6 strain SV-39 was the most closely related. The whole-genome phylogenetic tree was consistent with the phylogenetic analysis of amino acid sequences of the *DNApol* and *IVa2* trees (Figures 3, 4).

**Figure 5:**
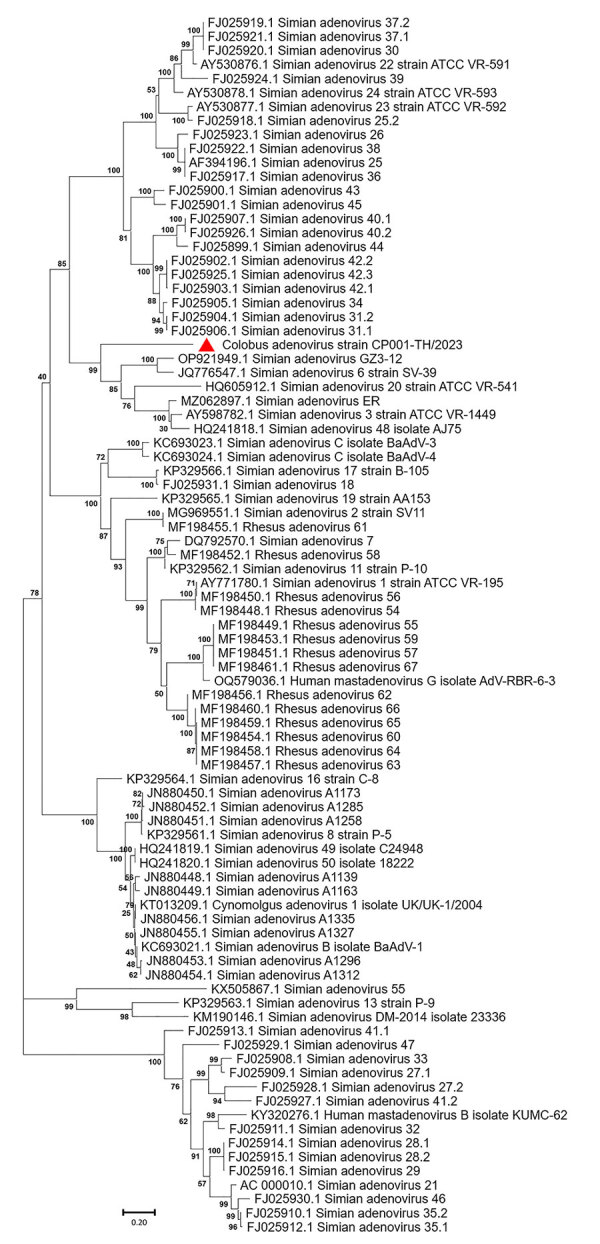
Phylogenetic analysis of amino acid sequences of *fiber* gene of novel mastadenovirus infection causing pneumonia in imported black-and-white colobuses (*Colobus guereza*), Thailand. Comparative analysis of the genome from this study, tentatively name colobus adenovirus (CoAdV), with various adenoviruses identified in nonhuman primates. Bootstrap values are shown at each node. Red triangle indicates the CoAdV CP001 TH/2023 identified in this study. GenBank accession numbers are provided. Scale bar indicates the number of substitutions per site. AdV, adenovirus; BaAdV, baboon adenovirus.

**Figure 6:**
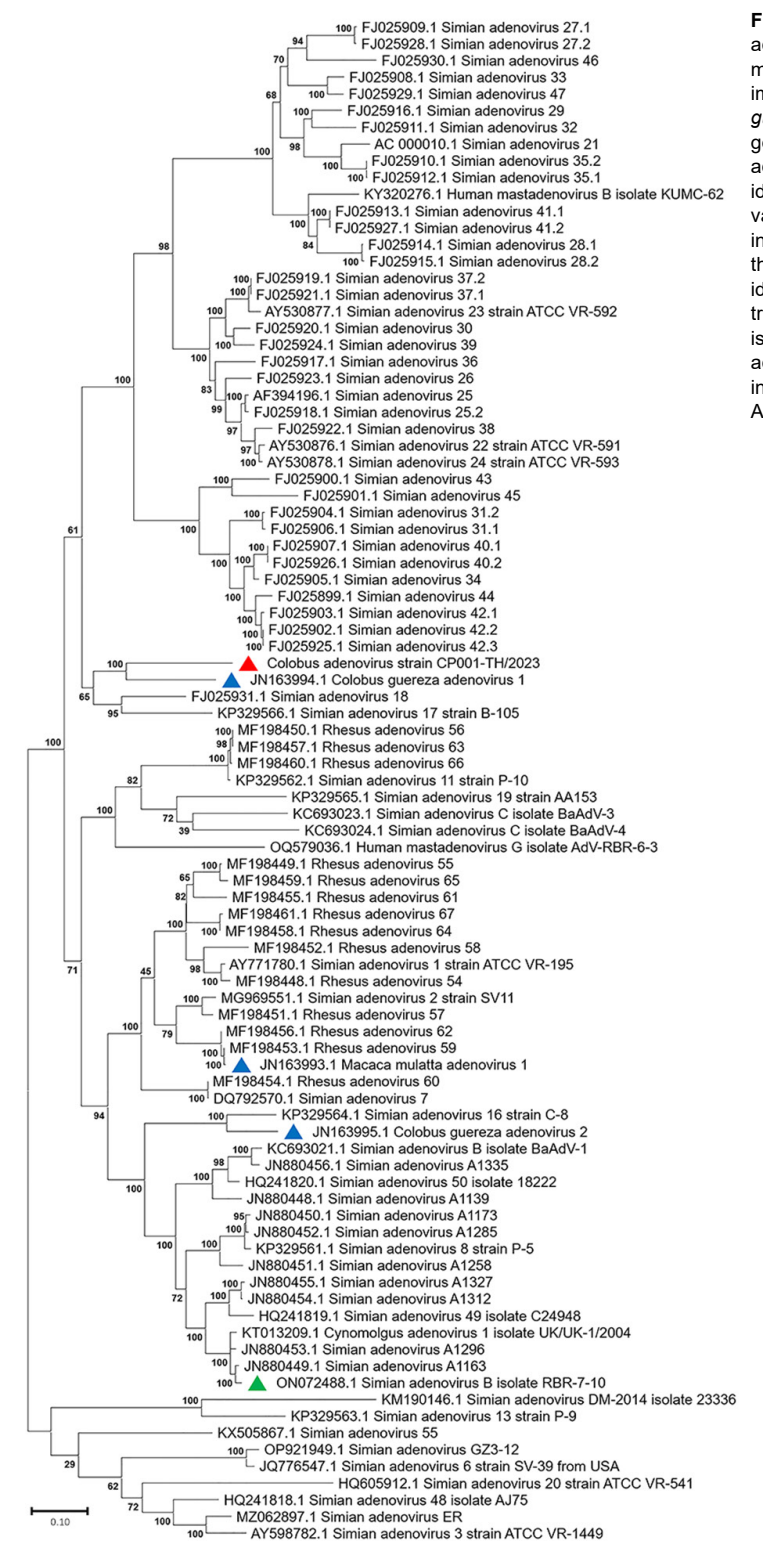
Phylogenetic analysis of amino acid sequences of *hexon* gene of novel mastadenovirus infection causing pneumonia in imported black-and-white colobuses (*Colobus guereza*), Thailand. Comparative analysis of the genome from this study, tentatively name colobus adenovirus (CoAdV), with various adenoviruses identified in nonhuman primates. Bootstrap values are shown at each node. Red triangle indicates the CoAdV CP001 TH/2023 identified in this study; blue triangles indicate AdVs previously identified in colobuses in Germany in 2011; green triangle indicates previously identified adenovirus isolated from macaques in Thailand. GenBank accession numbers are provided. Scale bar indicates the number of substitutions per site. AdV, adenovirus; BaAdV, baboon adenovirus.

Because limited information was available on other genes of previously described AdVs identified in colobuses in Germany, we were only able to analyze relatedness in the *fiber* gene (Figure 5). CoAdV CP001-TH/2023 shared 71.75%–80.50% nucleotide similarity with AdVs identified in colobuses in Germany in 2011, and *Colobus guereza* adenovirus 1 (GenBank accession no. JN163994) was the most closely related strain. Although the CoAdV *hexon* gene identified in this study genetically clustered closely with the *Colobus guereza* adenovirus 1 (GenBank accession no. JN163994) strain, it formed a distant phylogenic topology with 99% bootstrap support (Figure 6). However, CoAdV CP001-TH/2023 had a unique phylogenetic topology and did not cluster with other previously described AdVs identified in colobuses (GenBank accession nos. JN613993 and JN613995). Information for complete genome sequences of SAdV identified in Thailand was limited, and only the *fiber* gene was available. Thus, we also included the *fiber* gene of SAdV-B isolate RBR-7-10 (GenBank accession no. ON072488) to determine the phylogenetic relationship of the detected CoAdV; however, those sequences were distantly related (Figure 5). We did not identify any genetic recombination events within the CoAdV genome.

### CoAdV Pathology and Viral Distribution 

**Figure 7:**
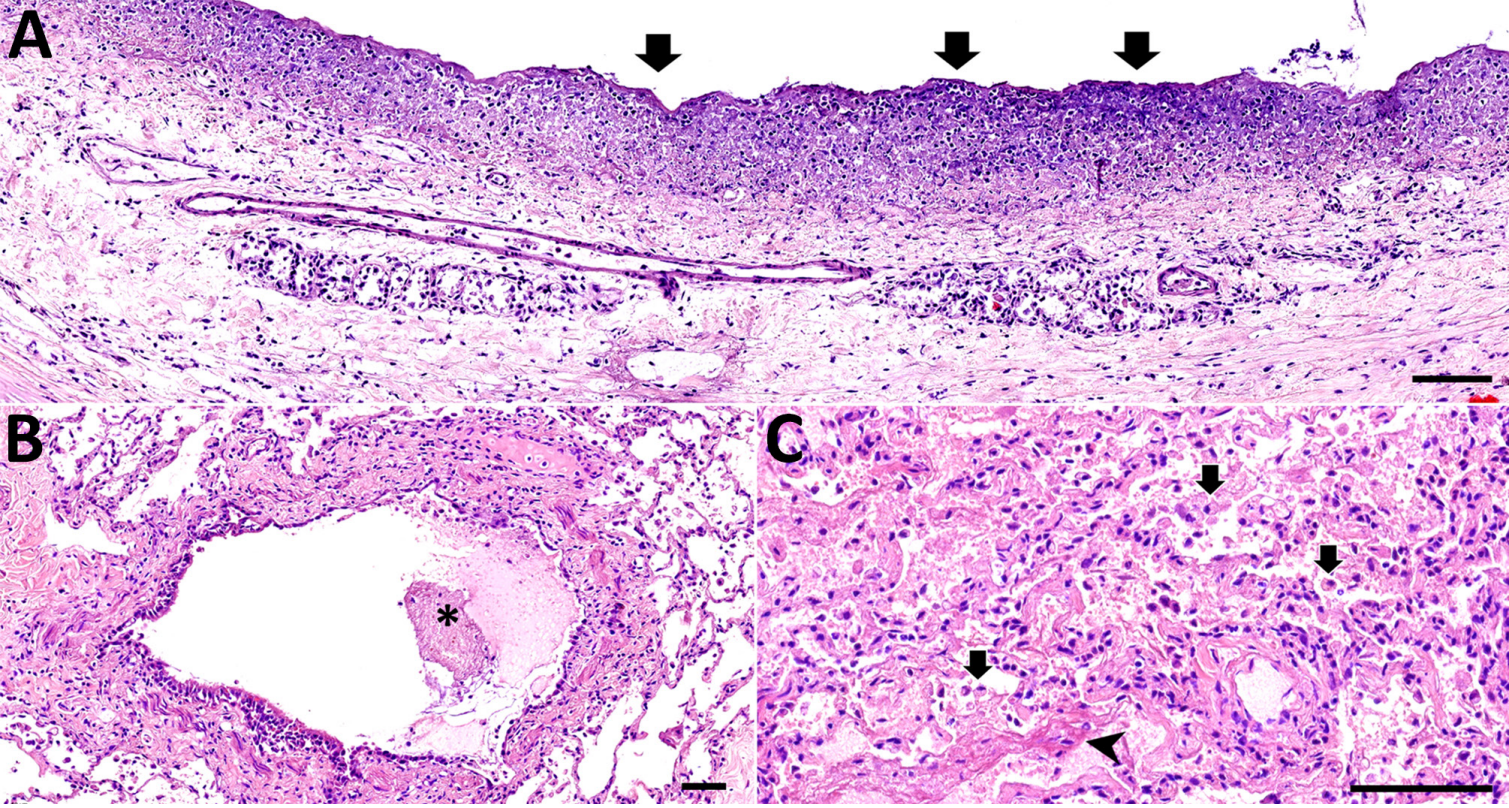
Hematoxylin and eosin–stained tissue samples from colobus 1 in an investigation of novel mastadenovirus infection causing pneumonia in imported black-and-white colobuses (*Colobus guereza*), Thailand. A) Trachea showing mucosa is extensively replaced by laminated bands of fibrillar material (arrows) intermixed with aggregates of karyorrhectic debris and degenerated neutrophils. B) Lung section showing lumen of the bronchial airways; flocculent mats of fibrin (asterisk) and neutrophils can be seen. C) Lung section showing alveoli filled with foamy macrophages (arrows), polymorphonuclear cells and eosinophilic material (arrowhead); alveolar capillaries are markedly engorged. Scale bars indicate 100 µm.

Overall, histologic findings were consistent across all colobuses and showed varying degrees of severity. The trachea mucosa was extensively replaced by laminated bands of eosinophilic fibrillar material mixed with scant aggregates of karyorrhectic debris. In addition, the tracheal lumen contained a small pool of neutrophils and karyorrhectic debris (Figure 7, panel A). The pulmonary interstitium was diffusely congested, with increased prominence and tortuosity of markedly engorged alveolar capillaries. In colobus no. 1, the pulmonary alveoli were multifocally filled with eosinophilic granular to homogenous material, intermixed with variable numbers of foamy macrophages, and fewer polymorphonuclear cells (Figure 7, panel B). Moreover, alveoli were overlaid by variable flocculent mats of eosinophilic fibrin. Similar mats of eosinophilic fibrin and a few neutrophils were seen in 1 lumen of the large bronchial airways (Figure 7, panel C). In colobus no. 2, we noted similar but milder changes.

**Figure 8:**
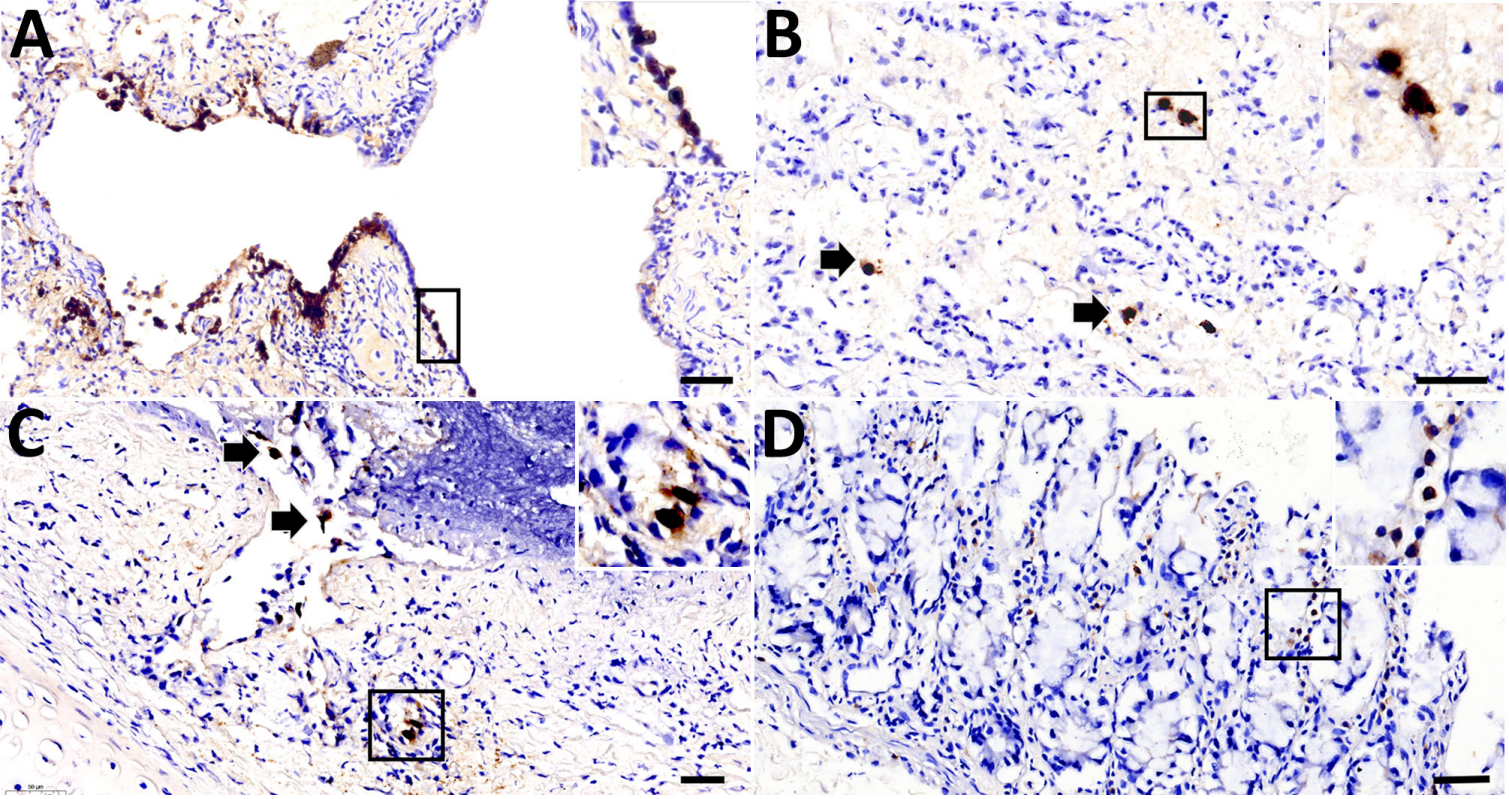
Adenoviral immunohistochemically stained lung and intestine sections from case 1 in an investigation novel mastadenovirus infection causing pneumonia in imported black-and-white colobuses (*Colobus guereza*), Thailand. A–C) Lung sections; D) intestine section. A) Nuclear labeling of adenoviral antigen in the bronchial epithelial lining; inset shows area of interest at 400× magnification. B) Adenoviral antigen is localized in the nuclei of pulmonary epithelial cells (arrows); inset shows endothelial-like cells from area of interest at 400× magnification. C) Infiltrated inflammatory cells and bronchial glands of the trachea (arrows); inset shows inflammatory cells at 400× magnification. D) Adenoviral antigen detected in rare, single inflammatory cells infiltrating the intestinal villi; inset shows area of interest at 400× magnification. Scale bars indicate 50 µm.

To determine the CoAdV distribution in tissue and localize the virus where lesions were located, we quantified the viral loads by using qPCR (Table). We primarily detected CoAdV in respiratory samples and noted the highest viral loads in the lung but also high loads in trachea tissue and oral and fecal swab samples. We found smaller amounts of the viral genome in the small intestine. We used IHC to determine CoAdV tissue localization, which revealed prominent positive immunoreactivity in the nuclei of bronchial epithelial cells (Figure 8, panel A), pulmonary alveolar macrophages, and endothelial cells of pulmonary capillaries (Figure 8, panel B). We also found local immunolabelling in the mucosal epithelial cells and glands of the trachea, particularly in areas that had pseudodiphtheritic membrane of necrosis (Figure 8, panel C). We identified IHC labeling in the small intestine (Figure 8, panel D) and in single lymphoid cells in mediastinal lymph node and tonsil.

### Viral Culture

**Figure 9:**
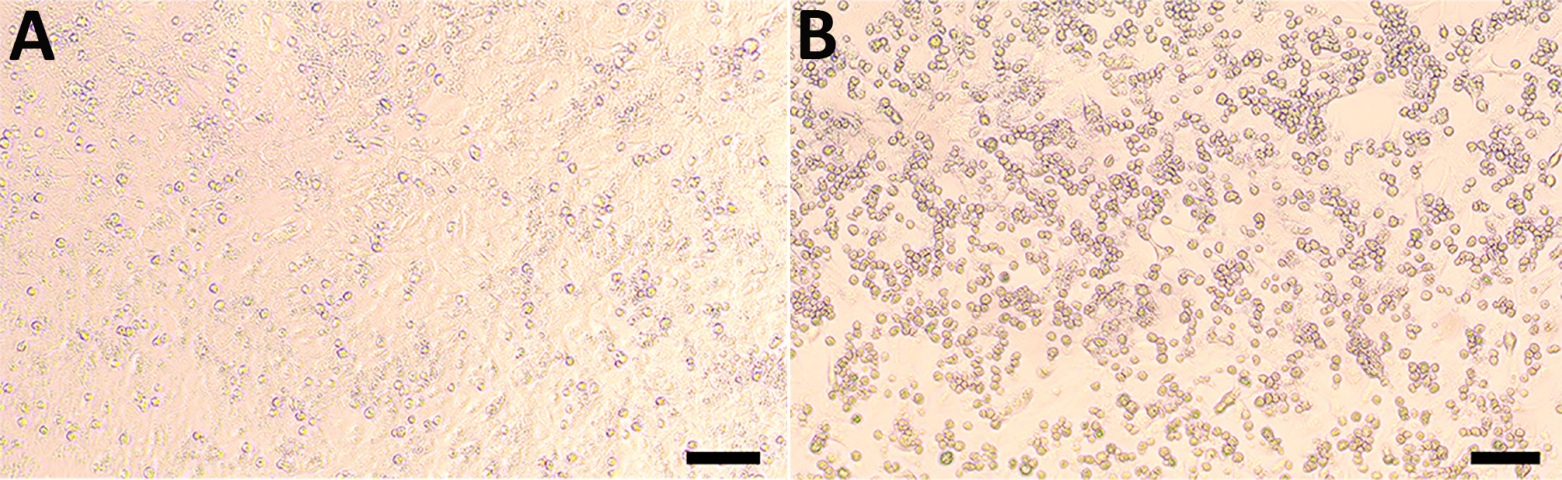
Vero cell isolation of novel mastadenovirus infection causing pneumonia in imported black-and-white colobuses (*Colobus guereza*), Thailand. Over 80% confluence of Vero cells were used for CoAdV isolation. A) Mock-infected cells. B) CoAdV-infected Vero cells at 5 days postinfection; clear cytopathic effects can be seen, characterized by cell rounding and detachment. Scale bars indicate 100 µm.

We successfully isolated CoAdV from fecal and oral swab samples of colobus nos. 6, 7, and 9 and from homogenized trachea and lung tissues of colobus no. 1. However, cell cultures inoculated with samples from colobuses 4 and 5 exhibited severe cytotoxicity, characterized by cell detachment and cell death at the first day postinoculation (dpi), leading us to discard those inoculations. Infected Vero cells revealed nonsyncytia, round-shaped CPE (Figure 9). The CPE first appeared at 3 dpi, characterized by cell rounding and detachment observed by 5 dpi. Subsequent infection of Vero cells with the supernatant from the primary virus isolation also resulted in similar CPE formation at 3 dpi. Conventional PCR confirmed the presence of CoAdV DNA in the infected cell culture supernatants. Sanger sequencing of the partial *DNApol* gene from cellular supernatant identified partial CoAdV sequences. We did not detect CoAdV DNA in mock-infected cells.

### In Situ Ultrastructure of CoAdV

**Figure 10:**
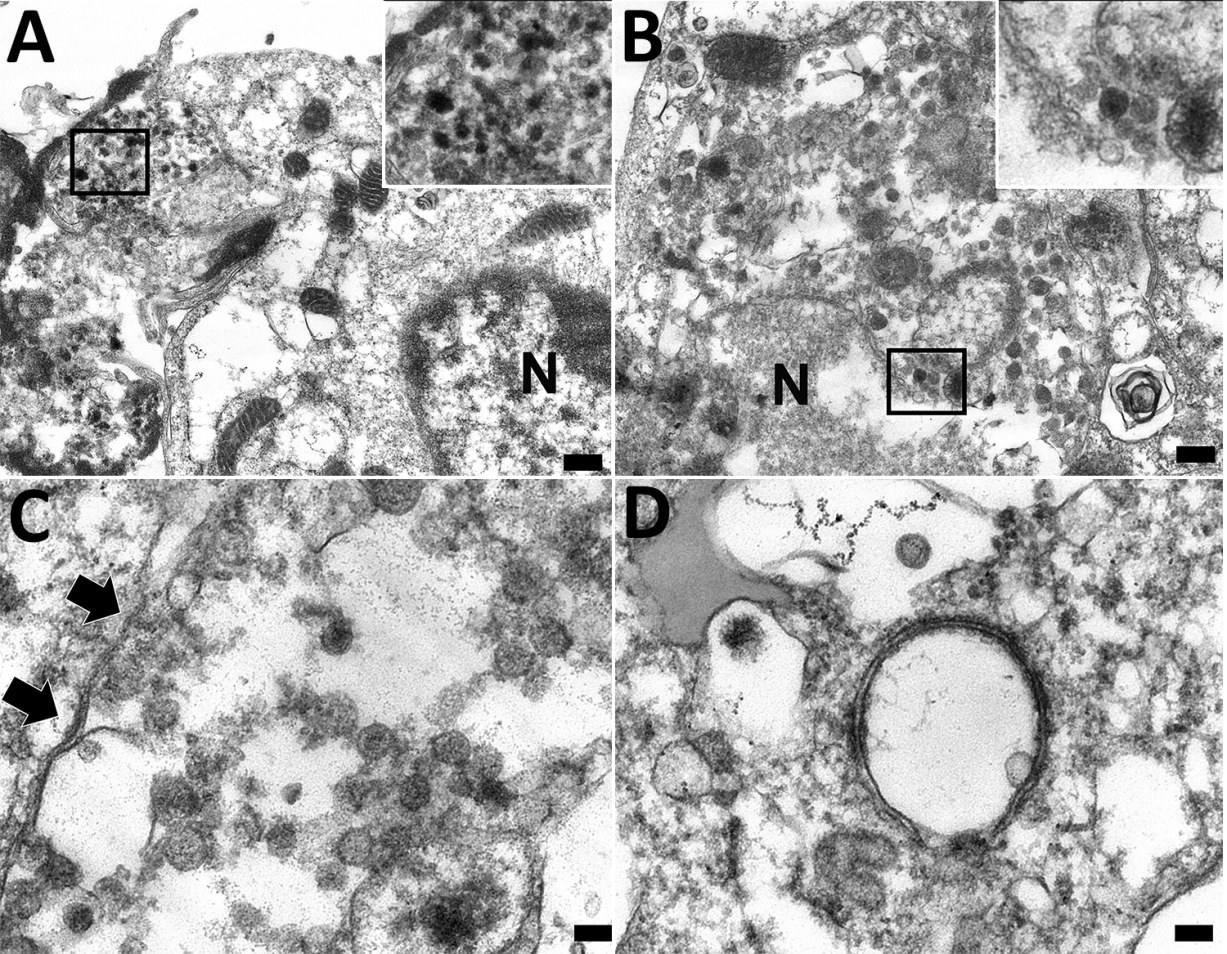
Transmission electron microscopy of formalin-fixed paraffin embedded lung section of case 1 in an investigation of novel mastadenovirus infection causing pneumonia in imported black-and-white colobuses (*Colobus guereza*), Thailand. A) Destructed bronchial epithelial cell showing degenerated plasma membrane and a large cytoplasmic vacuole containing electron-dense particles (inset area of interest at 100× magnification). N indicates nuclear membrane. Scale bar indicates 200 nm. B) Random viral particles distributed in cytosol; viral particles are seen near ruptured nuclear (N) membrane (inset area of interest at 100× magnification). Scale bar indicates 100 nm. C) Mature icosahedral electron-dense viral particles in the cytosol. Black arrows indicate plasma membrane. Scale bar indicates 100 nm. D) A vacuole containing electron-lucent particle and a free-living electron-dense particle in the cytosol. Scale bar indicates 100 nm.

**Figure 11:**
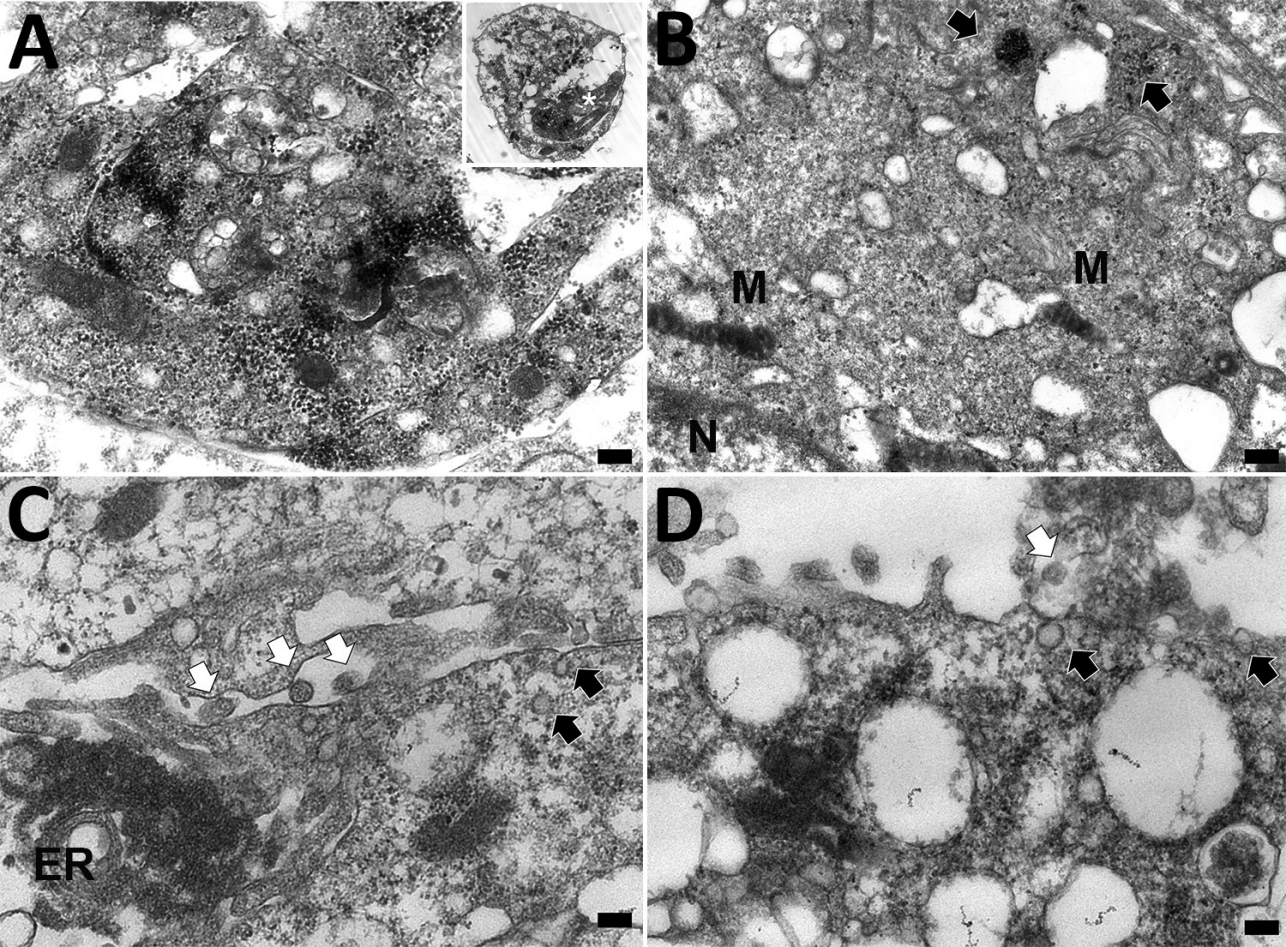
Transmission electron microscopy of Vero cell of case 1 in an investigation of novel mastadenovirus infection causing pneumonia in imported black-and-white colobuses (*Colobus guereza*), Thailand. A) Vero cells 1 day postinfection; B–C) Vero cells at 5 days postinfection A) Cytoplasmic vacuolated Vero cell contains numerous electron-dense particles packed within a destructed nucleus. Inset indicates the overall cellular morphology of an infected cell, and asterisk within indicates the area of higher magnification. Scale bar indicates 400 nm. B) Viral particles arranged within the cytosol as freely-distributed and clustered patterns (black arrows). M indicates mitochondria; N indicates nucleus. Scale bar indicates 300 nm. C) Electron-lucent viral particles within nuclear membrane and fused nuclear membrane (black arrows); electron-dense particles (white arrows) are seen within the endoplasmic reticulum (ER). Scale bar indicates 100 nm. D) A viral particle fused with plasma membrane (black arrow) and the budding virions (white arrow) in extracellular space. Scale bar indicates 100 nm.

Ultrastructural investigation of CoAdV-infected FFPE sections revealed pulmonary cellular damage by exhibiting rupture of cellular membrane, cytoplasmic vacuolization, and degenerated nuclear compartments. Within intact cells, condensed nuclear membrane contained various sizes of large cytoplasmic vacuolization containing numerous icosahedral electron-dense particles, each ≈80 nm in diameter, characteristic of adenoviruses (Figure 10, panel A). We considered those particles, typically seen attached near ruptured nuclear membranes or distributed randomly within the cytosol (Figure 10, panels B–D), of viral origin. Transmission electron microscopy analysis of CoAdV-infected Vero cells at 1 dpi showed prominent nuclear chromatin with early detection of viral virion within the nucleus (Figure 11, panel A). We also noted vesicles containing 80–90 nm icosahedral electron-dense or electron-lucent particles. We found cytoplasmic vacuolization and disruption of nuclear membranes in infected Vero cells at 5 dpi. We frequently observed numerous electron-dense particles in the cytosol. Virions were scattered throughout the cytosol and found within the endoplasmic reticulum or Golgi apparatus (Figure 11, panel B). In some infected cells, we observed mature virions fused with the cellular membrane being released into the extracellular space or passively through ruptured plasma membranes (Figure 11, panels C, D).

### Retrospective Study

We detected CoAdV nucleic acid in 4 oral and 2 rectal swab samples collected and archived from 4 of 7 colobuses that were imported into Thailand, exhibited respiratory distress, and were submitted for postmortem investigation during 2020–2022. Phylogenetic analysis of obtained sequences revealed that the sequences clustered together and were closely related to the CoAdV identified in this study (Appendix Figure). Investigation of archival samples derived from various other simian species were negative.

## Discussion

Using conventional PCR targeting the conserved *DNApol* gene of adenoviruses, we identified a novel mastadenovirus, CoAdV, in multiple black-and-white colobuses, a species currently listed as decreasing in population (*24*). Identification of this virus was associated with a fatal outbreak of respiratory disease. We propagated CoAdV in Vero cells and confirmed the virus via PCR and ultrastructural investigation. qPCR quantified viral loads in various organs, and immunohistochemistry and transmission electron microscopy revealed virus localization in tracheal epithelium and pulmonary parenchyma, associated with pneumonia. 

We successfully isolated CoAdV and characterized its complete genome sequence. Subsequent tissue analysis revealed that CoAdV infection was linked to respiratory lesions. Detection of CoAdV DNA and its localization in tissues with lesions suggests a major disease burden. Moreover, the identification of CoAdV in colobuses within the same household may indicate the virus’ contagious nature. Therefore, CoAdV infection should be considered a concern when pneumonia occurs in this species.

Adenovirus infection in colobuses has been reported previously (*9*), but no information regarding clinical relevance was provided in those cases, and only the partial *hexon* gene was characterized. Although phylogenetic analysis of the *hexon* gene revealed that our CoAdV CP001-TH/2023 is genetically close to *Colobus guereza* adenovirus 1 (GenBank accession no. JN163994), the virus we detected still exhibits diversity and shares less genomic and amino acid similarity with that virus. 

Infection associated with fatal outcomes in these colobuses cannot be definitively concluded without conducting animal challenge studies. The clinical severity of adenovirus infection in NHPs varies and often is associated with fatal diseases when infecting immunocompromised subjects (*4*). Although metagenomic studies in the fatal CoAdV cases we report did not indicate other underlying viral infection and results of ancillary testing were negative, the severe clinical outcomes observed suggest a potential role for stress-induced reactivation of a latent virus in the colobuses, exacerbated by transportation stress and possibly inadequate quarantine measures. In addition, the widespread severity of the illnesses raises the possibility that CoAdV might have originated from another animal source and was not native to this species. Although our phylogenetic analysis did not confirm direct links to viruses in related hosts, the adaptability of adenoviruses across species barriers supports that hypothesis (*6*,*7*,*10*,*11*).

To determine whether CoAdV is circulating or originating in Thailand, we made further attempts to investigate CoAdV infection in other NHPs; however, we could not identify this virus in samples from other NHPs, except in other colobuses that had respiratory problems or diarrhea. Further studies are needed to assess whether CoAdV is host-restricted because the infection appears potentially limited to colobuses. Detection of CoAdV in archival samples suggests the possibility of longstanding circulation within the colobus population. Supporting previous publications (*2*,*25*), the adenovirus identified in the colobuses was not genetically related to previously described adenoviruses in free-ranging macaques in Thailand. However, because we only investigated a small number of samples, definitive conclusions cannot be drawn. Future investigations incorporating serologic surveys could provide a more comprehensive assessment of the virus’ prevalence and transmission patterns. Such studies would be particularly valuable for determining whether CoAdV is circulating endemically or was recently introduced into Thailand. Because samples from close contact humans have not been investigated, the potential harmful effects of CoAdV infection to humans cannot be extrapolated. Reports of SAdV infection in non–natural hosts and humans, and subsequent transmission to other contracts (*6*), highlight the zoonotic transmission risk. Therefore, the spillover of CoAdV to susceptible hosts, including humans, should not be overlooked.

The CoAdV genome contains 32 putative coding sequences, including unique genes such as *V* and *IX*, and the *E1*, *E3*, and *E4* regions that are specific to the *Mastadenovirus* genus (*1*). CoAdV also has a single *fiber* gene, which is uncommon in related viruses. The <85% amino acid identity to its closest relative, SAdV-6 strain SV-39, and its identification in a new host support classifying CoAdV as a distinct species. Thus, we propose the virus as SAdV-J on the basis of species demarcation.

CoAdV phylogenetically clustered with most mastadenoviruses found in old-world monkeys, confirming unique clusters of adenoviruses within the Cercopithecidae family. Phylogenetic analysis of *DNApol* gene revealed that CoAdV shares genetic similarity with SAdV strain 55, identified in an endangered golden sub-nosed monkey (*Rhinopithecus roxellana*) in China, suggesting that CoAdV may have co-evolved with that viral SAdV species. Although the partial *hexon* gene of adenoviruses identified in colobuses in Germany was reported (*9*), our phylogenetic analysis of the *hexon* gene indicated that CoAdV is not related to those former strains. Given the unique phylogenic topology of CoAdV and the fact that interspecies recombination of mastadenoviruses has been previously reported (*8*,*9*), we investigated the potential recombination events. However, we did not identify any recombination breakpoints within the CoAdV genome. Although we successfully characterized a complete genome sequence of CoAdV, we acknowledge that the genomic analysis was limited to that single sequence. Future studies should aim to include multiple genome sequences to enhance our understanding of the genetic diversity and epidemiology of CoAdV.

Colobuses, native to Africa, are now experiencing a population decline (*24*). Despite extensive efforts to protect this species from becoming endangered, CoAdV infection causing fatal disease poses a possible threat. The transmission of CoAdV to native monkeys has not been elucidated, and the natural host of this virus remains unidentified, although imported infected reservoirs may spread the infection.

In conclusion, our study demonstrates infection with a novel virus, CoAdV, associated with respiratory disease in imported black-and-white colobuses and describes the isolation and characterization of this virus. CoAdV was identified in all colobus contacts, indicating its transmissibility. Genetic analysis of the isolated virus revealed that CoAdV is distantly related to other previously described SAdVs and appears to be a novel species in the genus *Mastadenovirus.* Although we did not find CoAdV in retrospective samples obtained from other NHPs, the potential for infection in NHPs and humans remains unclear. Further studies are needed to determine the virus’ role in disease development and host diversity. Extensive AdV surveillance in various animal species is crucial for elucidating the origins and spread of CoAdV in colobuses.

Appendix Additional information on novel mastadenovirus infection as cause of pneumonia in imported black-and-white colobuses (*Colobus guereza*), Thailand.
