# Supplementary material for: Novel Mastadenovirus Infection as Cause of Pneumonia in Imported Black-and-White Colobuses (Colobus guereza), Thailand
Source: Emerg Infect Dis. 2024 Dec;30(12):2544–58. doi: 10.3201/eid3012.241042 (PMC11616658; doi:10.3201/eid3012.241042)
Supplement: Appendix — Additional information on novel mastadenovirus infection as cause of pneumonia in imported black-and-white colobuses (Colobus guereza), Thailand. [file 24-1042-Techapp-s1.pdf]

Article DOI: https://doi.org/10.3201/eid3012.241042

EID cannot ensure accessibility for supplementary materials supplied by authors. Readers who have difficulty accessing supplementary content should contact the authors for assistance.

Novel Mastadenovirus Infection as Cause of Pneumonia in Imported Black-and-White Colobuses (*Colobus guereza*), Thailand

Appendix

Supplementary Methods

High-throughput and De Novo Sequencing

Briefly, 1 μg of extracted genomic DNA was randomly fragmented by sonication and subjected to 5′ phosphorylation and ligation with adaptors. AxyPrep Mag PCR Clean-up (Axygen, ThermoFisher Scientific Inc., Waltham, MA, USA) was used for size selection of adaptor-ligated DNA, recovering fragments ≈410 bp in length (with an insert size of around 350 bp). Each sample underwent PCR amplification for 8 cycles using P5 and P7 primers, designed for flow cell annealing in bridge PCR, with the P7 primer also carrying a six-base index for multiplexing. PCR products were then purified using AxyPrep Mag PCR Clean-up (Axygen), validated using Agilent 2100 Bioanalyzer (Agilent Technologies, Palo Alto, CA, USA), and quantified using Qubit2.0 Fluorometer (Invitrogen, Carlsbad, CA, USA). Libraries with different indexes were subsequently multiplexed and loaded onto Illumina HiSeq instrument following manufacturer's instructions (Illumina, San Diego, CA, USA). Sequencing was carried out in a 2x150 paired-end (PE) configuration, with image analysis and base calling conducting using HiSeq Control Software (HCS) + RTA 2.7 (Illumina). Raw sequences underwent quality control, including adaptor and primer sequence trimming using FASTP program (*1*), and quality assessment using FASTQC (*2*) and MultiQC tools (*3*). Cleaned sequences were aligned against the host genome using Bowtie2 (*4*), and sequence mapped to the host genome were processed and extracted using Samtools (*5*) and Bedtools (*6*). Sequences that did not map to the host genome underwent taxonomic profiling using Kraken2 (*7*) for short reads, followed by assembly

into longer sequences using SPADes (*8*) with a minimum contig size of 300 bps. All assembled contigs were screened for potential virus sequences through BLASTN homology-search against NCBI RefSeq virus sequences (downloaded as of July 2023). Contigs identified as virus-hit were analyzed for Open Reading Frames (ORFs) using orfipy (*9*). Functional annotation of predicted ORFs was performed by BLASTP against the UniRef90 database. The assembled AdV sequence underwent analysis by comparing nucleotide and protein identities to other published AdVs in the GenBank database using BLASTn and BLASTx algorithms, respectively. The sequence was further submitted to identify potential ORFs using ORFfinder on the NCBI platform.

Phylogenetic and Recombination Analysis

Phylogenetic analysis using maximum likelihood was performed with the *General Time Reversible* (GTR) model across lineages and a gamma (+Γ) distribution of rates across sites (GTR+G+I) as the best-fit model of nucleotide substitution for whole genome, *DNA pol*, and *fiber* genes. For the *IVa2* gene phylogenetic analysis, the Tamura-Nei 93 model with a gamma (+Γ) distribution of rates across sites (TN93+G+I) was chosen based on the Bayesian Information Criterion (BIC). All phylogenetic trees were subjected to 1,000 bootstrapping replicates, and the phylogenetic tree was constructed using MEGA X. Pairwise similarity between sequences was calculated using BioEdit 7. Furthermore, amino acid sequences of various genes, including *DNApol*, *IVa2*, *hexon* and *fiber* genes, were compared, and phylogenetic trees were constructed using the settings and protocols described above.

To detect putative recombination events in CoAdV, the alignment of all published SAdVs was analyzed using Recombination Detection Program (RDP) version 4.101, following the settings and analysis criteria reported in previous studies (*10–12*). The potential recombination breakpoints identified by RDP were further analyzed using similarity and bootscan analysis in the SIMPLOT software package version 3.5.1.

Viral Load Quantification and Cellular Localization

CoAdV viral loads in various organs were determined using SYBR-based quantitative PCR. Briefly, 5 µl of extracted nucleic acids were added to a 20 µl reaction master mix containing 200 nM of each primer and 2.5 mM of $MgCl_2$. The qPCR reaction was performed in a Rotor-Gene Q real-time PCR cycler (Qiagen GmbH, Hilden, Germany) with cycling conditions of 40 cycles at 95°C for 3s and 60°C for 20s for annealing and fluorescence acquisition. A

melting curve analysis was subsequently conducted from 72°C to 85°C to validate the amplification specificity. Amplification detected after 38 cycles of qPCR was considered negative. CoAdV cellular localization was determined by immunohistochemistry (IHC). Briefly, IHC analysis was performed on the Leica Microsystems Bond maX System (Leica Microsystems, IL, USA). The slides were deparaffinized by incubating at 72°C for 30 min and treated with Bond Dewax Solution (Leica Microsystems). Antigen retrieval was achieved by incubating the sections in Bond Epitope Retrieval Solution 2 (Leica Microsystems) at 37°C for 10 min. The IHC procedure used the Bond Polymer Refine Detection kit (Leica Microsystems), which employs a three-step indirect immune-peroxidase technique. The primary antibody against AdV clone 2/6 and 20/11 (MAB805; Chemicon, IL, USA) was applied at a 1:1500 dilution for 40 min at room temperature. After three consecutive washing with Bond Wash Solution (Leica Microsystems), sections were treated with 3% (v/v) H_2O_2 for 5 min and rinsed again three times with Bond Wash Solution. Next, the Post Primary Polymer (Leica Microsystems) was applied for 12 min, followed by incubating with the Polymer Poly-HRP anti-Mouse/Rabbit IgG (PowerVision Poly-HRP IHC Detection Systems, Leica Biosystems) for 12 min, with washing steps in between. The 3,3′-Diaminobenzidine (DAB) chromogen was then applied for 3 min to visualize the reaction, followed by rinsing with dH_2O. Slides were finally counterstained with hematoxylin. For controls, an HAdV-infected human lung section served as a positive control, while CoAdV-positive lung sections were incubated with normal rabbit IgG NI01 (Sigma-Aldrich, South Africa) as a negative control.

Viral Culture

The cells were cultured in Minimum Essential Medium (MEM) supplemented with 10% fetal bovine serum (FBS), 1× Antibiotic-Antimycotic solution, and 1× GlutaMAX (GIBCO, Thermo Fisher Scientific, Waltham, MA, USA) until reaching 80%–90% confluency. Oral and rectal swabs from diseased colobuses (nos. 4–6, 7, and 9) collected in viral transport medium, as well as supernatants from lung and trachea homogenized in PBS from deceased colobus (no. 1), were filtered through a 0.45 μm pore-sized membrane using a syringe filter. The filtrates were diluted 1:2 in viral growth medium (VGM; MEM supplemented with 2% FBS, 1× Antibiotic-Antimycotic solution, and 1× GlutaMAX), and 200 μL of each dilution was inoculated onto the Vero cell monolayer. The inoculum was allowed to adsorb to the cell monolayer at 37°C under a 5% CO_2 atmosphere for 1 h. Following adsorption, 800 μL of VGM was added to each well, and

the cells were further incubated at 37°C under a 5% CO_2. Two blind passages were performed, and cytopathic effects (CPE) were monitored daily for 5 days. Supernatants from wells showing CPE were collected and stored at –80°C for subsequent AdV-specific PCR confirmation, following the protocol described earlier. Additionally, cell pellets were harvested by centrifugation at 200 *x g* for 5 min, washed three times with PBS, and fixed in a 2.5% glutaraldehyde buffered solution. The harvested pellets were initially fixed at room temperature for 10 min, and then kept at 4°C overnight. The fixed cell pellets were prepared for TEM analysis.

Transmission Electron Microscopy

The CoAdV-positive FFPE lung sections and cell pellets obtained from viral isolations were subjected to ultrastructural investigation using TEM. For FFPE sections, the 4-µm sections were marked in areas presenting IHC reactions using an alcohol-xylene resistant pen under microscopy. The sections were then stained with 2% (v/v) osmium tetroxide (OsO_4) and subjected to pre-TEM processing using modified pop-off techniques (*13*,*14*). For cell pellets, the samples were triple washed with 0.1 M PBS and incubated with 1% (*v*/v) OsO_4 for 1 h. The cells were then dehydrated in acetone, embedded in epoxy resin, thick- sectioned, and stained with toluidine blue to determine cellular quality. The selected areas were marked, trimmed, ultrathin sectioned at 80 nm, and placed on a copper grid. The samples were examined under a TEM machine (HT7800, Hitachi, Tokyo, Japan) operating at 80 kV.

Reference

1. Chen S. Ultrafast one-pass FASTQ data preprocessing, quality control, and deduplication using fastp. iMeta. 2023;2:e107. PubMed https://doi.org/10.1002/imt2.107

2. Andrews S. FastQC: A quality control tool for high throughput sequence data 2010 [cited 2024 Jun 16]. https://github.com/s-andrews/FastQC

3. Ewels P, Magnusson M, Lundin S, Käller M. MultiQC: summarize analysis results for multiple tools and samples in a single report. Bioinformatics. 2016;32:3047–8. PubMed https://doi.org/10.1093/bioinformatics/btw354

4. Langmead B, Wilks C, Antonescu V, Charles R. Scaling read aligners to hundreds of threads on general-purpose processors. Bioinformatics. 2019;35:421–32. PubMed https://doi.org/10.1093/bioinformatics/bty648

5. Danecek P, Bonfield JK, Liddle J, Marshall J, Ohan V, Pollard MO, et al. Twelve years of SAMtools and BCFtools. Gigascience. 2021;10:giab008. PubMed https://doi.org/10.1093/gigascience/giab008

6. Quinlan AR, Hall IM. BEDTools: a flexible suite of utilities for comparing genomic features. Bioinformatics. 2010;26:841–2. PubMed https://doi.org/10.1093/bioinformatics/btq033

7. Wood DE, Lu J, Langmead B. Improved metagenomic analysis with Kraken 2. Genome Biol. 2019;20:257. PubMed https://doi.org/10.1186/s13059-019-1891-0

8. Prjibelski A, Antipov D, Meleshko D, Lapidus A, Korobeynikov A. Using SPAdes de novo assembler. Curr Protoc Bioinformatics. 2020;70:e102. PubMed https://doi.org/10.1002/cpbi.102

9. Singh U, Wurtele ES. orfipy: A fast and flexible tool for extracting ORFs. Bioinformatics. 2021;37:3019–20. PubMed https://doi.org/10.1093/bioinformatics/btab090

10. Piewbang C, Jo WK, Puff C, van der Vries E, Kesdangsakonwut S, Rungsipipat A, et al. Novel canine circovirus strains from Thailand: evidence for genetic recombination. Sci Rep. 2018;8:7524. PubMed https://doi.org/10.1038/s41598-018-25936-1

11. Piewbang C, Techangamsuwan S. Phylogenetic evidence of a novel lineage of canine pneumovirus and a naturally recombinant strain isolated from dogs with respiratory illness in Thailand. BMC Vet Res. 2019;15:300. PubMed https://doi.org/10.1186/s12917-019-2035-1

12. Piewbang C, Kasantikul T, Pringproa K, Techangamsuwan S. Feline bocavirus-1 associated with outbreaks of hemorrhagic enteritis in household cats: potential first evidence of a pathological role, viral tropism and natural genetic recombination. Sci Rep. 2019;9:16367. PubMed https://doi.org/10.1038/s41598-019-52902-2

13. Piewbang C, Chaiyasak S, Kongmakee P, Sanannu S, Khotapat P, Ratthanophart J, et al. Feline morbillivirus infection associated eith tubulointerstitial nephritis in black leopards (*Panthera pardus*). Vet Pathol. 2020;57:871–9. PubMed https://doi.org/10.1177/0300985820948820

14. Piewbang C, Wardhani SW, Chanseanroj J, Yostawonkul J, Boonrungsiman S, Saengkrit N, et al. Natural infection of parvovirus in wild fishing cats (*Prionailurus viverrinus*) reveals extant viral localization in kidneys. PLoS One. 2021;16:e0247266. PubMed https://doi.org/10.1371/journal.pone.0247266

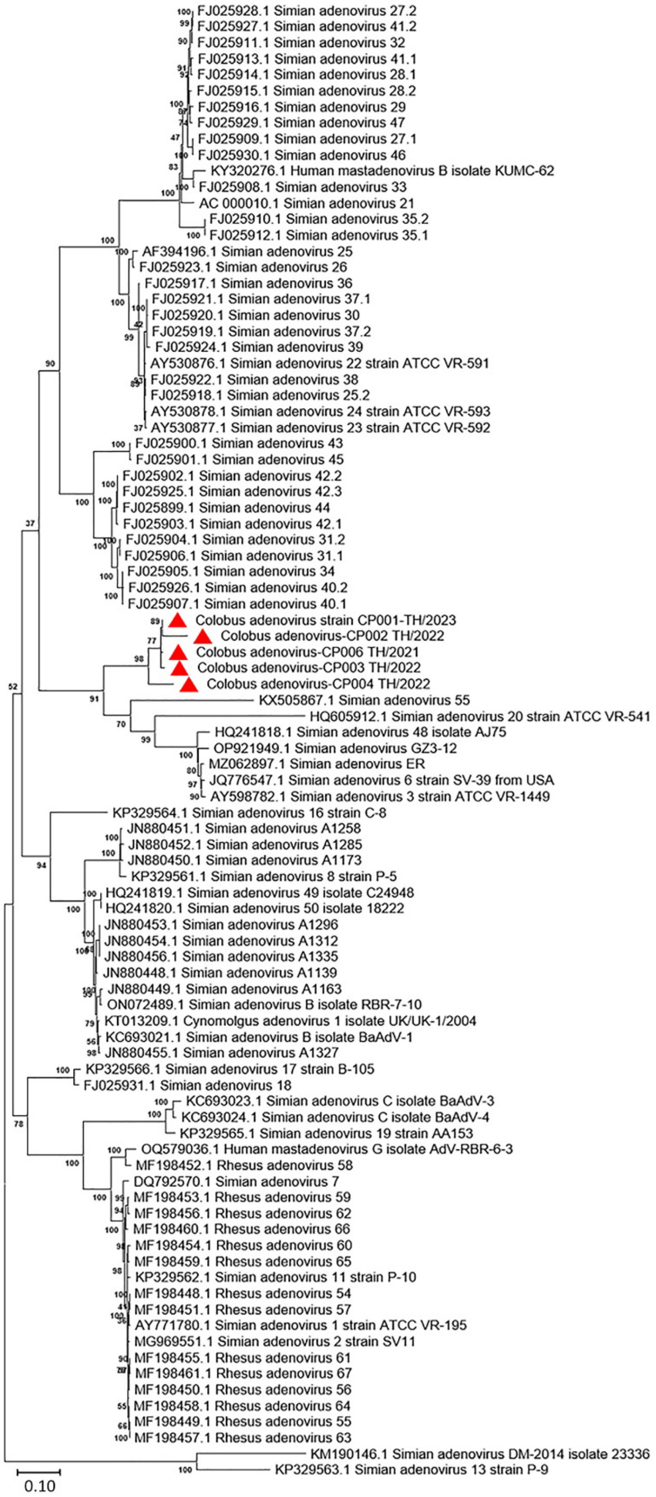

Appendix Figure. Phylogenetic analysis of CoAdV *DNA pol* gene. Comparative analysis of the CoAdV genomes with various adenoviruses identified in NHPs was conducted based on partial amino acid sequences of *DNA pol* gene. Red triangles indicate the CoAdV identifying in this study. Bootstrap values are shown at each node, and the bars indicate the number of substitutions per site.
